# Healthcare Expenditure and Economic Performance: Insights From the United States Data

**DOI:** 10.3389/fpubh.2020.00156

**Published:** 2020-05-13

**Authors:** Viju Raghupathi, Wullianallur Raghupathi

**Affiliations:** ^1^Koppelman School of Business, Brooklyn College of the City University of New York, Brooklyn, NY, United States; ^2^Gabelli School of Business, Fordham University, New York, NY, United States

**Keywords:** healthcare, economic performance, personal healthcare expenditure, hospital expenditure, GDP, visual analytics

## Abstract

This research explores the association of public health expenditure with economic performance across the United States. Healthcare expenditure can result in better provision of health opportunities, which can strengthen human capital and improve the productivity, thereby contributing to economic performance. It is therefore important to assess the phenomenon of healthcare spending in a country. Using visual analytics, we collected economic and health data from the Bureau of Economic Analysis and the Bureau of Labor Statistics for the years 2003–2014. The overall results strongly suggest a positive correlation between healthcare expenditure and the economic indicators of income, GDP, and labor productivity. While healthcare expenditure is negatively associated with multi-factor productivity, it is positively associated with the indicators of labor productivity, personal spending, and GDP. The study shows that an increase in healthcare expenditure has a positive relationship with economic performance. There are also variations across states that justify further research. Building on this and prior research, policy implications include that the good health of citizens indeed results in overall better economy. Therefore, investing carefully in various healthcare aspects would boost income, GDP, and productivity, and alleviate poverty. In light of these potential benefits, universal access to healthcare is something that warrants further research. Also, research can be done in countries with single-payer systems to see if a link to productivity exists there. The results support arguments against our current healthcare system's structure in a limited way.

## Introduction and Background

Healthcare spending and the impact that it has on economic performance are important considerations in an economy. Some studies have shown that improvements in health can lead to an increase in Gross Domestic Product (GDP) and vice versa (1–3). Healthcare holds a significant place in the quality of human capital. The increased expenditure in healthcare increases the productivity of human capital, thus making a positive contribution to economic growth (4, 5). However, there is ongoing debate on what kinds of healthcare spending and what level of optimal spending is beneficial for economic development (6–8).

The theory of welfare economics is relevant to the current research. Welfare economics is a branch that deals with economic and social welfare by analyzing how the resources of the economy are allocated among the social agents (9, 10). Here, we analyze the allocation of resources in terms of spending within the healthcare sector and assess its influence on economic welfare. In addition to this, we draw from several related studies in laying a strong foundation for our research. The relationship between health and economic growth has been examined extensively across multiple studies (11–16). Based on a study that examined the impact of health on economic growth in developing countries, it was evident that a decrease in birth rates positively affected economic growth (17). During the period of study, health expenditures rose threefold, from $83M to $286M, and outpaced growth in GDP. The study showed that health and income mutually affected each other and concluded that problems affecting healthcare delivery caused negative impact on economic growth (18). Arora (19) investigated the effects of health on economic growth for industrialized countries and found a strong association. In a study of the impact of health indicators for the period 1965–1990 for developed and developing countries, economic performance in developing countries increased significantly with an improvement in public health (20). Studies have proposed that an annual improvement of 1 year in life expectancy increases economic growth by 4% (1, 21). Similarly, another study in 2001 emphasized that the existence of a healthy population may be more important than education, for human capital in the long term (22). Examining 21 African countries for the 1961–1995 period and 23 Organization for Economic Cooperation and Development (OECD) countries for the 1975–1994 period with the extended Solow growth model, authors found that 23 OECD health stocks affect growth rate of per capita income (23). Muysken (24) also investigated whether health is one of the determinants of economic growth and concluded that an iterative relationship exists between economic growth and health—high economic growth leads to investments in human capital and to health advancement, and good population health leads to more labor productivity and economic growth. Aghion et al. (25) utilized the *Schumpeterian growth theory* to analyze channels associated with the influence of national health on economic growth. The theory emphasizes the importance of maternal and child health on the critical dimensions of human capital. Another element that has been shown to be a critical element for sustainable economic growth is high life expectancy (26). Aghion et al. (27) applied the endogenous growth theory, which proposes that a better life expectancy enhances growth, to analyze the relationship between health and economic growth. The study examined life expectancy for various ages in OECD countries and concluded that a decline in mortality rates for the age groups below 40 has the effect of increasing economic growth Aghion et al. (27).

Based on the above-mentioned studies, we surmise that higher income per capita is associated not only with life expectancy, but also with numerous other measures of health status. While health is not the only indicator of economic development—indeed, we need to consider the impact of other factors, such as education, political freedom, gender, and many other social attributes (1, 3, 28)—health is definitely an integral non-income component that should be considered in a measure of economic development. People generally give high priority and value to a long and healthy life (2, 25). Secondly, the rate of achievement of this goal to aspire for a long and healthy life differs widely across countries (11, 13, 29). The Human Development Index, in addition to suggesting a correlation between income and health, also expresses a strong correlation between an individual's place in the income distribution and his or her health outcomes within a country (2, 30). This within-country correlation is particularly strong in developing countries. In comparing the growth of income with improvements in health outcomes, it is common to account for simultaneous causation. As an example, people who are healthy have the ability to be more productive in school and at work, reflecting that good health can be a precursor for better economic development (4). Additionally, a higher income allows individuals or governments to make investments that yield better health (28). Finally, differences in the quality of education, government, health, and other institutions across countries, in human capital, or in the level of technology can induce correlated movements in health and income (16). One also needs to account for the dynamic effects built into many of the potential causal outlets. For example, improvements in health may only result in increased worker productivity after a lag of several decades. Similarly, when life expectancy rises, there can be increases in population growth that may temporarily reduce income per capita (31).

The per capita health expenditures of countries vary in terms of economic development.

Whereas, high-income countries spend, on average on healthcare, $3,000 on each citizen, low-income countries only spend up to $30 per capita. It is also important to consider healthcare expenditure expressed as a percentage of GDP (5, 14). While some countries spend higher than 12% of GDP on healthcare, others spend as little as 3% (32). There are at least two methods that can explain the association between a country's healthcare expenditure and economic performance. In the first scenario, healthcare expenditure is considered an investment in human capital. Human capital accumulation is then perceived to be a source of economic growth (e.g., via increased productivity). Therefore, an increase in healthcare expenditure is likely to be associated with a higher GDP (30, 33). In the second scenario, an increase in healthcare expenditure can lead to regular health interventions (e.g., annual medical-checkups, preventive screening, etc.), which are likely to improve labor and productivity; this, in turn, will increase the GDP (34). Both these mechanisms reflect an iterative phenomenon between healthcare and GDP. Nevertheless, the relationship needs to be checked for endogeneity—which we aim to study in this research.

An important dimension in the relationship between health expenditure and economic performance is the factor of the productivity of workers. In developed countries, labor is scarce, and capital is abundant as a factor of production (2, 31, 35). But this situation is reversed in developing countries where economic growth and economies are based on labor. Here, an increase in individuals' poor health will likely lead to a loss in labor workforce and productivity (4, 16). Therefore, addressing public health and health expenditures, though important for both developed and developing countries, is more critical for the latter (3, 4, 11, 13, 16, 36). It is generally assumed from common knowledge that individuals who are healthier are able to work more effectively, in terms of physical and mental workload. Also, adults who were healthier as children will have acquired more human capital in the form of education, which is explained by the proximate effect of health on the level of income (37). Simultaneously, the impact of individual income on health is also important (38, 39). Higher income can result in better health by facilitating access to better nutrition, preventative treatment, good sanitation, safe water, and affordable quality healthcare. Additionally, health can also be a cause of high income, by allowing individuals to work more, be more productive and earn higher income during the lifetime (35).

The impact of health on education is an important factor that plays a role in healthcare expenditure and economic performance (30, 33). Children who enjoy good health can attend school regularly and have the potential of high learning ability and cognitive development. Also, if good health continues through adulthood, it will enable the population to recover the investments in education (30, 33, 39).

Another significant dimension in the relationship that healthcare spending has with economic development is the impact of health on savings. Good health can increase the life expectancy and encourage an individual's motivation to have savings (such as for retirement) and to make more business investments, both of which are beneficial activities for economic performance (1). Population health is an important healthcare component whose impact should be considered. A healthy population can reduce the expense on national healthcare and increase the potential for earnings. In this manner, the economic impact of population health can occur at the micro and macro levels (1, 2, 4, 5). It is no surprise that some countries assign a higher value to gains from health than gains from income (36, 40–43). Additionally, most countries have witnessed an increase in life expectancy despite a persistent income gap over the last 50 years (44), reflecting the monetary benefits that can accrue from investing in healthcare (2, 44).

In this research, we acknowledge the significance of healthcare expenditure and analyze its association with the economic performance. We conduct the analysis at a national level for the United States using the data from the Bureau of Economic Analysis (BEA) and the Bureau of Labor Statistics (BLS). We incorporate the techniques of visual and descriptive analytics (45–47). Our findings provide insight on the differences in health spending and economic performance across the various states of the U.S. The research offers implications for governments 2008; and national policy makers to identify dimensions of healthcare that contribute to national economic performance. It is especially important for policy that addresses population health issues of a nation.

The rest of the paper is organized as follows: section Research 2 describes the methodology; section 3 presents the analyses and results; section 4 contains a discussion of results with implications; section 5 offers the scope and limitations of the research; and finally, section 6 presents the conclusions.

## Research Methodology

### Data Collection and Variables

We analyze state-level data and ascertain patterns that offer insight into the healthcare spending and economic performance of various states in the United States. Our methodology includes the stages of data collection and variable selection, data preparation, analytics platform and tool selection, and analytics implementation. We collected economic and health data from the Centers for Medicare and Medicaid Services (CMS) (https://www.cms.gov/Research-Statistics-Data-and-Systems/Statistics-Trends-and-Reports/NationalHealthExpendData), Bureau of Economic Analysis (BEA) (https://www.bea.gov/iTable/iTable.cfm?reqid=70&step=1&isuri=1&acrdn=2#reqid=70&step=1&isuri=1), and the Bureau of Labor Statistics (BLS) (https://www.bls.gov/lpc/data.htm; https://www.bls.gov/webapps/legacy/tusa_1tab1.htm) for a period of 12 years (2003–2014). The variables relate to various economic performance and healthcare spending indicators. Table 1 shows the variables in the research.

**Table 1:** List of variables.

**Category**	**Variable**	**Description**
Economic performance	Percentage change in multifactor productivity (MFP) (%)	Measure of economic performance that compares the amount of goods and services produced to the amount of combined inputs used to produce those goods and services.
	Average weekly hours worked (#)	The total number of hours worked over a specified period of time, divided by the total number of weeks worked in the time period.
	Average hours/day spent purchasing goods/services (#)	The total number of daily hours spent purchasing goods and services
	Labor productivity (index)	The efficiency at which labor hours are utilized in producing output of goods/services measured as output per hour of labor.
	Total hours worked	The total number of hours worked by wage/salary workers, unpaid family workers and unincorporated self-employed workers to produce output.
	Per capita GDP ($)	The total output produced by an industry or sector, which is measured as the industry or sector's sales or receipts plus commodity taxes and changes in inventories, divided by population.
	Per capita personal income ($)	The average income earned per person in a given area in a specified year calculated by dividing the area's income by its population.
Healthcare expenditure	Per capita drugs expenditure ($)	Estimates of expenditures for prescription drugs, including retail sales of human-use, dosage-form drugs, biological drugs, and diagnostic products that are available only by a prescription.
	Per capita health expenditure ($)	Expenditures in the National health expenditure accounts represent aggregate health care spending in the U.S. divided by total population.
	Per capita home health ($)	Covers medical care provided in the home by freestanding home health agencies (HHAs). Medical equipment sales or rentals not billed through HHAs and non-medical types of home care are excluded.
	Per capita hospital expenditure ($)	Covers all services provided by hospitals to patients. These include room and board, ancillary charges, services of resident physicians, inpatient pharmacy, hospital-based nursing home and home health care, and any other services billed by hospitals in the United States.
	Per capita nursing ($)	Covers nursing and rehabilitative services provided in freestanding nursing home facilities. These services are generally provided for an extended period of time by practical nurses and other staff.
	Per capita other professional service ($)	This category includes spending for Medicaid home and community-based waivers, care provided in residential care facilities, ambulance services, school health, and worksite health care.
	Per capita personal healthcare ($)	Personal Health Care (PHC) comprises all of the medical goods and services that are rendered to treat or prevent a specific disease or condition in a specific person. These include hospital care; professional services; other health, residential, and personal care; home health care; nursing care facilities and continuing care retirement communities; and the retail outlet sales of medical products
	Per capita physician ($)	Covers services provided in establishments operated by Doctor of Medicine (M.D.) and Doctors of Osteopathy (D.O.), outpatient care centers, plus the portion of medical laboratories services that are billed independently by the laboratories.
Control variables	Population	The population used in the NHEA tables is defined as the U.S. Census resident population plus the net undercount.
	State/region	Name of the state/region
	Year	Year

The data was analyzed using the business intelligence tool Tableau for visualization, R programming language for regression analysis, and SPSS Modeler for neural network analysis.

### Visual Analytics Method

We utilize visual analytics to analyze healthcare spending and economic performance data. With visual analytics, one can discover patterns and relationships that are unexpected, and get timely and rational assessments of the phenomenon that is being analyzed (46, 48). Descriptive analytics, as a technique in visual analytics, helps one understand past and current trends and make informed decisions in a domain (48). By deploying this approach, we take a more data-driven approach to understanding the trends and associations between healthcare expenditure and economic performance scenario.

The technology of analytics is used increasingly in the domain of healthcare. As a business intelligence component, analytics allows statistical and quantitative analyses of large data repositories, enabling evidenced-based decision making (49). Specifically, in the domain of healthcare, analytics offers timely, relevant and quality information that can help healthcare entities and governments optimize health resource allocation goals effectively (50).

We deploy visual analytics based on the belief that it offers an effective tool to comprehend healthcare expenditure at a national level and analyze its impact on economic performance. We now discuss the results of our analyses in the following section.

## Analyses and Results

We analyzed the data for patterns and relationships between the indicators of healthcare spending and economic performance. Healthcare expenditure refers to aggregate healthcare spending in an economy, including expenditure relating to hospitals, home health agencies, prescription drugs, nursing facilities, and personal healthcare.

### Distribution of Hospital Expenditure Per Capita by Hospitals

**Figure 1:**
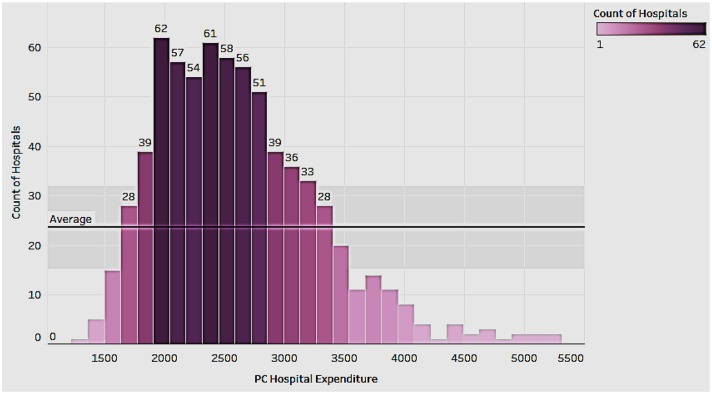
Per capita hospital expenditure distribution.

To get an idea of the state of hospital expenditure we looked at the distribution of expenditure by hospitals in the country (Figure 1). Hospital expenditure includes all service provided to patients, including room, ancillary charges, physician services, in-patient pharmacy services, and nursing home and home care. In Figure 1, the intensity of color of the bars depicts the number of hospitals such that the darker the color, the higher the number of hospitals with the expenditure. Clearly, the distribution is right-skewed. While the majority of the hospital expenditures per capita rank between $1,600 and $3,500, there are several outliers on the right side. Additionally, even though per capita hospital expenditure on average is within $3,500, there are still some hospitals where the average cost is higher.

### Hospital Expenditure Per Capita and GDP Per Capita by State

**Figure 2:**
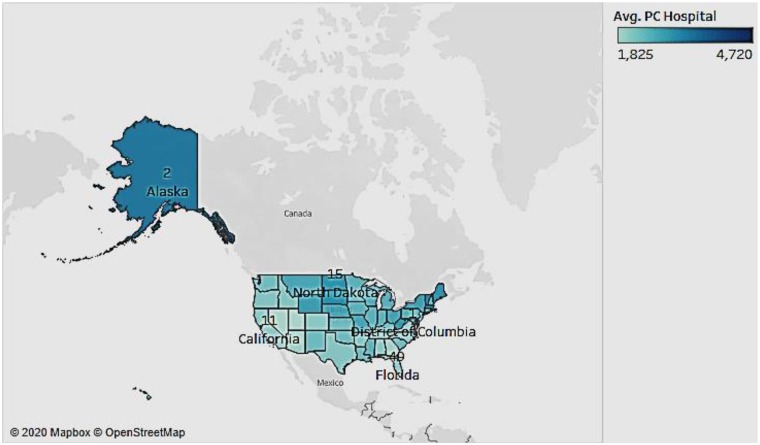
Per capita hospital expenditures and per capita GDP rank by state.

We now looked to see if there was any association between the hospital expenditure per capita and the GDP rank of the state (Figure 2). The figure depicts the per capita hospital expenditures by the intensity of the color (the darker the color, the higher the expenditures), and the state rank in terms of GDP per capita as a label in the state. We see that progressive states such as California with a high GDP rank have lower per person hospital expenditure; Nevada has a higher GDP rank than South Dakota but has a lower per capita hospital expenditure. In fact, the hospital expenditure in South Dakota is almost double that of Nevada. This suggests that the states that have higher economic performance (GDP) have legislative and innovative measures that support healthcare research, thereby resulting in lowered costs to the patients.

### Population and Per Capita Healthcare Expenditure

**Figure 3:**
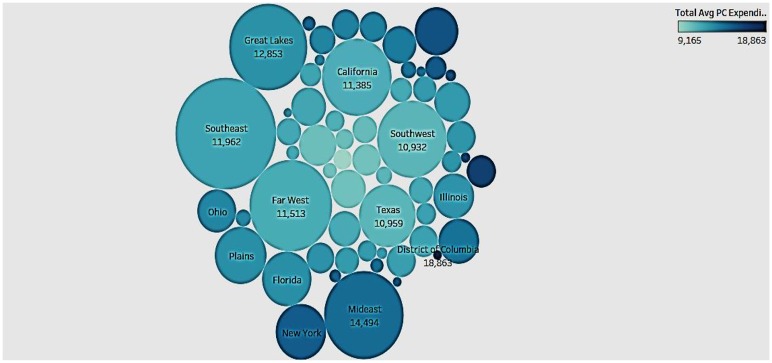
Overview of population size and total per capita healthcare expenditure.

Having compared the healthcare expenditure of a state with its GDP, we now wanted to see if there was any association with the population of a state (Figure 3). In the bubble chart the size depicts the population of the state and the color depicts the healthcare expenditure (darker colors represent higher expenditures). Interestingly, we see that sparsely populated states such as District of Columbia (DC) have higher healthcare spending than densely populated states like Texas. On the other hand, states like New York have high population and high expenditure. Therefore, there appears to be no correlation between population size and total average per capita expenditure, proving that population qualifies as a control variable in our dataset.

### Association of Hospital Expenditure With GDP Per Capita and Changes in Multifactor Productivity Over Time

**Figure 4:**
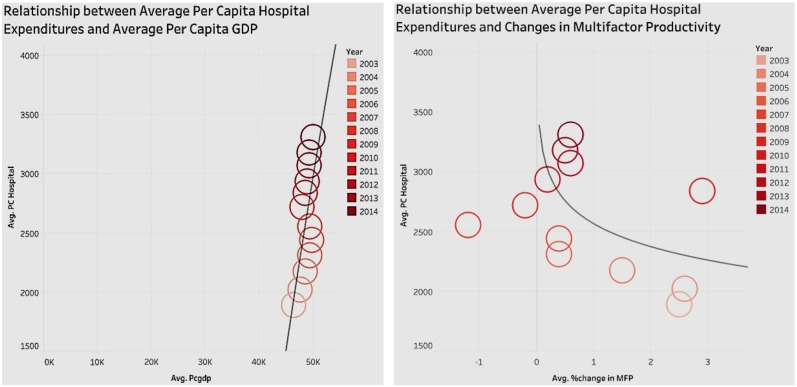
Relationship of hospital expenditures with per capita GDP, and changes in multifactor productivity.

We wanted to study the pattern of growth of hospital expenditure with GDP and with changes in multifactor productivity, from 2003 to 2014 (Figure 4). Both associations are shown side by side in Figure 4.

In Figure 4, the circles represent the performance for a year, with the intensity of the color indicating the recency of the year. In terms of the graph showing average per capita GDP and average per capita hospital expenditure, we see that since 2003, as the average per capita GDP increases, so does the per capita hospital expenditure. The positive correlation between the average per capita GDP and average per capita hospital expenditure implies that, by proxy, healthcare has a positive effect on GDP (economic performance).

The other graph in Figure 4 shows the relationship of Multifactor Productivity (MFP) with hospital expenditure. MFP is a measure of economic performance that reflects the overall efficiency with which inputs are used to produce outputs. Figure 4 shows that since 2003, the average per capita hospital expenditure has been increasing, but there is no obvious pattern in association with the changes in multifactor productivity. Also, it is worth noting that the trend line shows that there is a slight negative correlation between the changes in multifactor productivity and average per capita hospital expenditure.

### Association of Personal Healthcare Costs With Average Hours Per Day Spent on Purchasing Goods and Services, and With Changes in Multifactor Productivity (MFP)

**Figure 5:**
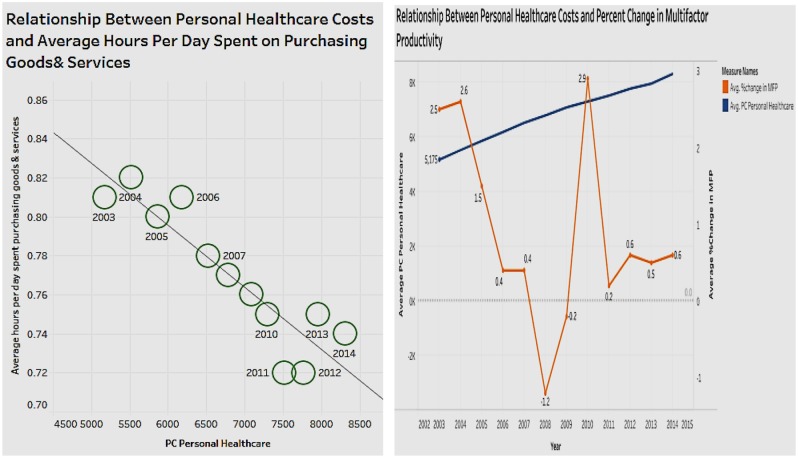
Relationship of personal healthcare costs with average hours per day spent on purchasing goods and services, and changes in multifactor productivity.

Personal healthcare expenditure determines the out-of-pocket costs incurred by the population. Figure 5 represents two associations of hospital expenditure side by side—with general purchases of the population, and with changes in MFP. In the association of hospital expenditure with general purchases of the population, we estimated the purchasing power of the population using the average hours spent per day on purchasing goods and services. The figure shows a negative relationship such that as personal healthcare costs increase, the average time spent on purchases declines. This is because as personal healthcare costs increase, the amount of available money for spending decreases, affecting the time spent on buying goods and services. Figure 5 also shows the association between hospital expenditure and changes in MFP. The line chart/trend line in the figure indicates that there is no obvious correlation between personal healthcare costs and percent change in MFP. This is consistent with the analysis of hospital expenditure which also had no association with MFP. One can infer that that a change in healthcare costs does not affect the economic cycle.

### Association of Healthcare Expenditure With Per Capita Personal Income

**Figure 6:**
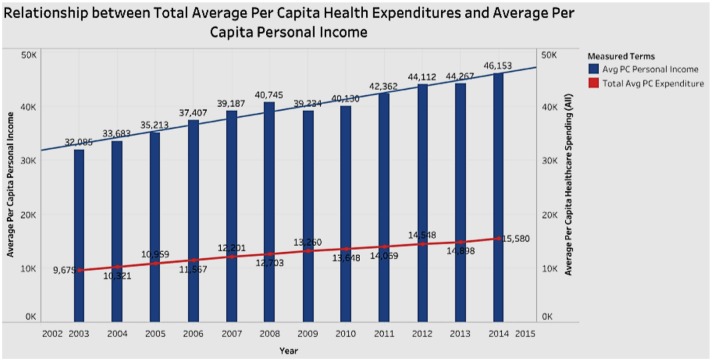
Association between per capita healthcare spending and personal income.

In looking for associations between healthcare expenditure and personal income (Figure 6) we see that between 2003 and 2014, personal income mostly increased while total healthcare spending has increased as a percentage of income. This confirms two trends—Americans spend more on healthcare over time; and personal income increases faster than that of healthcare expenditure in terms of dollar amount.

### Association of Hospital and Physician Expenditures With Labor Productivity

**Figure 7:**
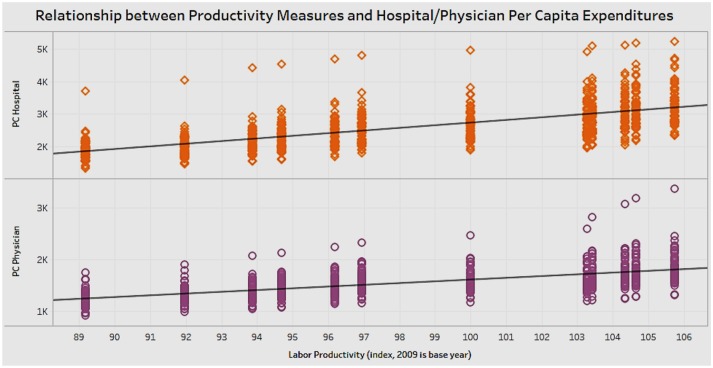
Correlation between labor productivity and hospital and physician expenditures.

Physician expenditure and hospital expenditure are components of overall healthcare costs of a state. We wanted to analyze if there was any association of labor productivity with physician expenditure and hospital expenditure (Figure 7). The scatterplot in the figure shows that spending in physician or hospital costs is positively correlated with an increase in labor productivity. It appears that healthcare spending has a positive relationship with labor productivity in the United States.

### Association of Per Capita Healthcare Expenditure With Labor Productivity and With GDP

**Figure 8:**
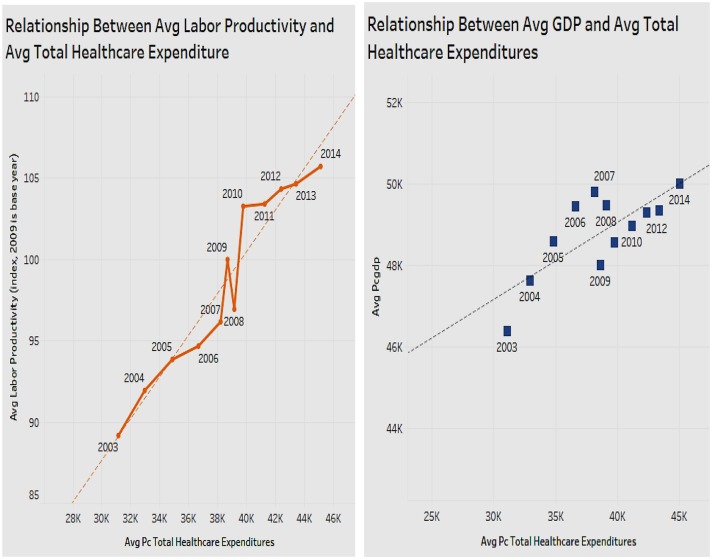
Relationship between total per capita healthcare expenditures and labor productivity.

In terms of healthcare expenditure, the above analysis revealed that physician and hospital expenditure were positively associated with labor productivity. We next explored if total healthcare expenditure which is an aggregate of all components is also associated with labor productivity, and with per capita GDP, both shown side by side (Figure 8). The figure shows that as the total healthcare expenditure increases, labor productivity also increases. There is a positive correlation between total per capita healthcare expenditure and labor productivity. Thus, by increasing healthcare expenditure, the health status of Americans will improve, increasing labor productivity. Figure 8 also shows the association of total healthcare expenditure with an alternate measure of economic performance, namely the GDP. The figure depicts a chart with a trend line that shows that as total healthcare expenditures increase, GDP also increases. Healthcare expenditure of a state has a positive relationship with the GDP of the state.

### Associations Between Personal Healthcare Expenditure, Hospital Expenditure, Nursing Expenditure, and Average Weekly Hours Worked

**Figure 9:**
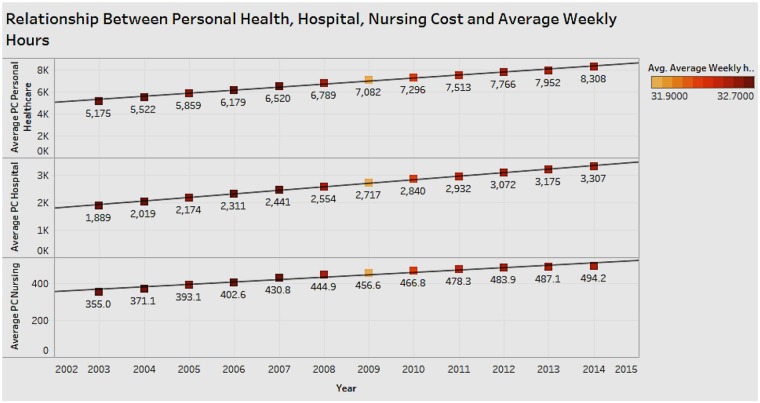
Relationship between personal health, hospital, nursing costs, and average weekly hours.

It is important to see the relationship between average hours worked (weekly) as a measure of economic performance and healthcare expenditure comprising personal healthcare, nursing, and hospital costs (Figure 9). From the figure we can see that as each of the health costs increases, there is no obvious change for average weekly hours. There appears to be no correlation between health costs and average weekly hours, which indicates there is no effect on productivity.

### Association of Personal Healthcare Expenditure With Per Capita GDP

**Figure 10:**
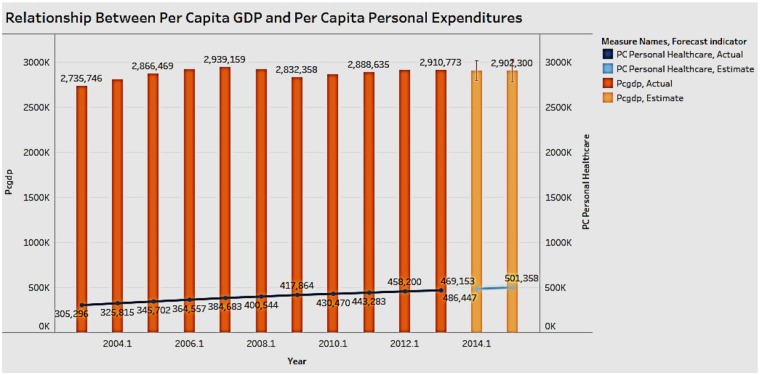
Correlation between per capita personal healthcare expenditure and per capita GDP.

Figure 10 shows the association between personal healthcare expenditure and GDP per capita.

In the figure the bar graph depicts the GDP and the trend line represents the personal healthcare expenditure. The last 2 years, which have a lighter color, represent the forecasted result. The chart shows that personal expenditure costs have steadily risen over the years, while the GDP does not show large fluctuations. A correlation is hard to establish between personal healthcare costs and GDP; it is possible that there may be extraneous types of healthcare expenditure that have an influence on the GDP.

### Distribution of Various Types of Healthcare Expenditures Across Years

**Figure 11:**
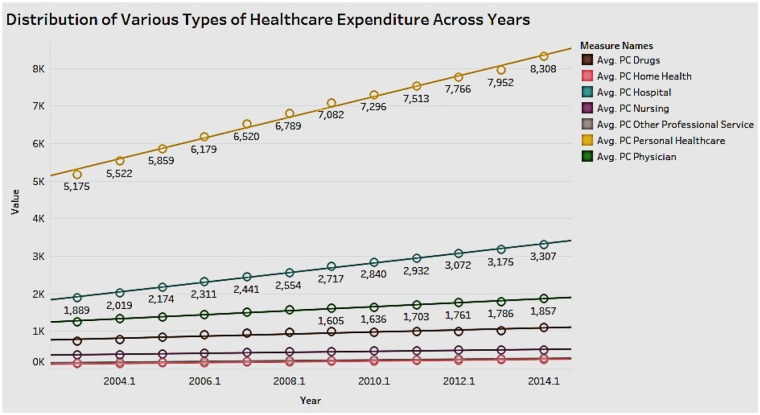
Distribution of various types of healthcare expenditures across years.

It is important to explore the different types of healthcare expenditure and their distribution over the years (Figure 11). Personal healthcare expenditure (includes private and public insurance) has the highest average of the types of spending in the years 2003 to 2014. This is followed by hospital and physician expenditure. The rise in personal healthcare expenditure has led to a high demand for reasonably priced private health insurance across the United States. The government needs to increase the affordability of public insurance to increase the reach and benefit more people.

### Association Between Personal Healthcare Expenditure Per Capita and Total Hours Worked

**Figure 12:**
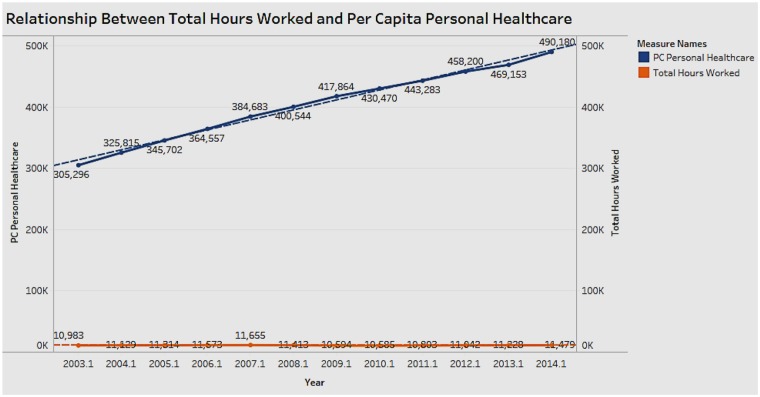
Relationship between hours worked and per capita personal healthcare expenditure.

Figure 12 shows the relationship between personal healthcare expenditure and total hours worked for the years 2003 to 2014. The growth of expenditure costs is not proportional to the rate of change in working hours. There appears to be no correlation between expenditure and working hours; however, from the other analyses, we know that healthcare expenditure has a positive correlation with income.

### Association Between Personal Healthcare Expenditure and Other Personal Expenditure

**Figure 13:**
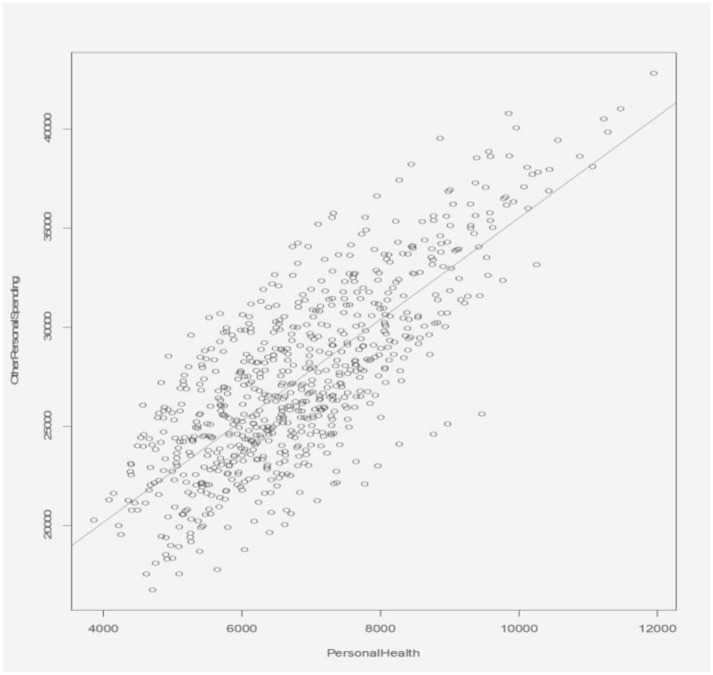
Relationship between personal health expenditure and other personal expenditure.

The relationship between personal healthcare expenditure and other personal expenditure is shown in Figure 13. The scatterplot shows the personal health expenditure having a positive correlation with the other personal expenditure. The ratio between them basically stays the same, which shows that an increase in personal health care expenditure does not impose a burden, significant enough to cause a reduction in other personal spending.

### Important Healthcare Expenditure Predictors of Per Capita GDP

**Figure 14:**
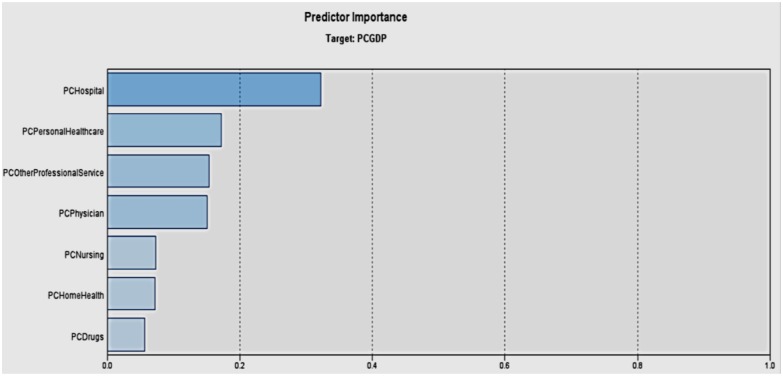
Importance of healthcare expenditure predictors for per capita GDP.

We wanted to explore which type of healthcare expenditure has the most significant influence on GDP. Figure 14 shows a machine learning based neural network model to analyze which type of healthcare spending affects the per capita GDP the most. The bars indicate to what extent the associated variable is determined by the target variable, namely per capita GDP. Among the different types of healthcare spending, hospital expenditure affects the per capita GDP the most, followed by personal healthcare. It confirms the fact that the effect of healthcare spending in the different care areas will have differential effects on the economy.

## Discussion

Our research offers several important findings that have implications for policy. While healthcare expenditure is negatively associated with multi-factor productivity, it is positively associated with labor productivity, personal spending, and GDP. However, this is not a causal relationship, and our inference is limited. Nevertheless, the research establishes, within the scope of the study, that an increase in healthcare expenditure has a positive relationship with economic performance. There are also variations across states that justify further research. Building on this and prior research, policy implications include that the good health of citizens indeed results in overall better economy. Therefore, investing carefully in various healthcare aspects would boost income, GDP, and productivity, and alleviate poverty. In light of these potential benefits, universal access to healthcare is something that warrants further research. Also, research can be done in countries with single-payer systems to see if a link to productivity exists there. Our results support arguments against our current healthcare system's structure in a limited way.

## Scope And Limitations

Our research has a few limitations. First, economic events such as recession may affect the validity of our results. Also, this research uses several proxies for productivity. Ideally, we should also track the hours of time spent being sick, which will affect both attendance and productivity; however due to unavailability of data this was not feasible. This research studies the data at a state level while other studies may drill down further to county and city level. Our research uses secondary data and is therefore subject to the limitations posed by the secondary source in terms of availability and veracity. Finally, the effects of healthcare spending on a different group (such as varying age groups) within a state were not studied. Nevertheless, the study offers a window into the relevance of healthcare expenditure in overall economic performance at a national level.

## Conclusions

Our findings suggest that, in general, there is a positive association between healthcare spending and the economic indicators of labor productivity, personal income, per capita GDP, and other spending. Also, personal healthcare spending adversely impacts time spent on purchases of goods and services. There is no association between healthcare spending and change in multi-factor productivity (MFP) or working hours. Different states require varied investment in personal health expenditure, even if they have the same level of labor productivity. Overall, the study contributes to the growing literature on healthcare expenditure and economic performance. It outlines how the government can allocate healthcare expenditure in key dimensions that can stimulate economic growth while also improving the well-being of the population. It is also critical that policy makers implement appropriate policies at the macroeconomic level—targeted at public health expenditure and economic development. Overall, in light of the potential benefits of healthcare to the economy, universal access to healthcare is an area that warrants further research.

## Data Availability Statement

Publicly available datasets were analyzed in this study. These can be found here: CMS; BEA; BLS; https://www.cms.gov/Research-Statistics-Data-and-Systems/Statistics-Trends-and-Reports/NationalHealthExpendData; https://www.bea.gov/iTable/iTable.cfm?reqid=70&step=1&isuri=1&acrdn=2#reqid=70&step=1&isuri=1; https://www.bls.gov/lpc/data.htm; https://www.bls.gov/webapps/legacy/tusa_1tab1.htm.

## Ethics Statement

Since this study uses aggregated national data, both ethical approval and written informed consent from the participants were not required for this study in accordance with the local legislation and institutional requirements.

## Author Contributions

VR and WR contributed equally to all parts of manuscript preparation and submission.

## Conflict of Interest

The authors declare that the research was conducted in the absence of any commercial or financial relationships that could be construed as a potential conflict of interest.
